# Outcomes of patients with suspected or confirmed melioidosis: A prospective multinational, multicenter, observational study

**DOI:** 10.1371/journal.pntd.0014562

**Published:** 2026-09-08

**Authors:** Koukeo Phommasone, Siriluck Anunnatsiri, Suwatthiya Kitsaran, Sue J. Lee, Pankham Vongphachanh, Ko Chang, George H. Talbot, Kevin O’Shea, Paul B. Eckburg, Stephen D. Prior, Elizabeth A. Ashley, Nicholas P. J. Day, Direk Limmathurotsakul

**Affiliations:** 1 Lao-Oxford-Mahosot Hospital-Wellcome Trust Research Unit, Microbiology Laboratory, Mahosot Hospital, Vientiane, Lao People's Democratic Republic; 2 Mahosot Hospital, Vientiane, Lao People's Democratic Republic; 3 Faculty of Medicine, Khon Kaen University, Khon Kaen, Thailand; 4 Department of Medicine, Sunpasitthiprasong Hospital, Ubon Ratchathani, Thailand; 5 Mahidol Oxford Tropical Medicine Research Unit, Faculty of Tropical Medicine, Mahidol University, Bangkok, Thailand; 6 Centre for Tropical Medicine and Global Health, Nuffield Department of Medicine, University of Oxford, Oxford, United Kingdom; 7 AN2 Therapeutics, Menlo Park, California, United States of America; 8 Department of Tropical Hygiene, Faculty of Tropical Medicine, Mahidol University, Bangkok, Thailand; Institut Pasteur, FRANCE

## Abstract

**Background:**

Melioidosis is an infectious disease caused by the Gram-negative bacterium *Burkholderia pseudomallei*. The disease is difficult to diagnose and treat. Here, we investigated outcomes of patients with suspected or confirmed melioidosis in Laos and Thailand.

**Methodology:**

We conducted a prospective, multinational, multicenter, observational study at Mahosot Hospital (MH) in Laos, and Srinagarind Hospital (SRH) and Sunpasitthiprasong Hospital (SUH) in Thailand. We enrolled adult patients (age ≥ 18 years) with suspected or confirmed melioidosis. For suspected-melioidosis cases, patients who had septic shock, had sepsis with at least one risk factor associated with melioidosis (including diabetes mellitus, chronic kidney disease, thalassemia major, malignancy and immunosuppressive therapy) or were prescribed ceftazidime or meropenem by attending physicians were enrolled, provided they were within 24 hours of hospitalization at the study hospitals. For confirmed-melioidosis cases, patients who had a clinical specimen tested positive for *B. pseudomallei* by either culture or antigen-detection immunofluorescence microscopy were enrolled. The primary endpoints were 28-day and 90-day mortality.

**Principal findings:**

From November 2023 to October 2024, 627 patients were screened and 200 patients were enrolled (43 for suspected-melioidosis and 157 for confirmed-melioidosis). Five of 43 patients enrolled with suspected-melioidosis (12%) were subsequently diagnosed with culture-confirmed melioidosis since enrollment. 28-day and 90-day mortality were 14% (6/43) and 23% (10/43) in suspected-melioidosis patients, respectively; and 25% (39/157) and 36% (57/157) in confirmed-melioidosis patients, respectively. In multivariable Cox regression models, higher Sequential Organ Failure Assessment (SOFA) score at enrollment and intensive care unit (ICU) admission on the day of enrollment were strongly associated with mortality outcomes. Admission to SRH and longer duration of symptoms prior to enrollment were independently associated with survival outcomes.

**Conclusions:**

Future clinical trials aiming to reduce mortality in patients with suspected or confirmed melioidosis should consider stratification based on the severity of organ dysfunction at enrollment. Differences in mortality between study sites may reflect residual confounding and should be carefully considered in sample size calculations.

## Author summary

Melioidosis is a frequently fatal infection caused by the soil bacterium *Burkholderia pseudomallei* and is a major cause of sepsis in Southeast Asia. Because the bacterium is intrinsically resistant to many commonly used antibiotics and melioidosis is difficult to diagnose using clinical presentation alone, clinicians often initiate empirical antibiotic therapy effective against *B. pseudomallei* once the disease is suspected. Treatment is continued for a complete course once the diagnosis is confirmed by bacterial culture results. However, patients treated for “suspected” melioidosis are seldom studied, and the factors associated with mortality in melioidosis remain poorly defined, in part because few studies have spanned more than one country. We prospectively followed 200 adults admitted with suspected or confirmed melioidosis at three hospitals in Laos and Thailand for 90 days. 90-day mortality was high in patients with both suspected and confirmed melioidosis. Factors associated with mortality included the severity of organ failure and intensive-care admission at enrollment, whereas a longer duration of symptoms before enrollment and care at one study hospital were associated with survival. These findings suggest that future trials aiming to reduce melioidosis mortality should account for organ-failure severity and between-hospital differences when designing patient selection and calculating sample size.

## Introduction

Melioidosis is a frequently fatal infectious disease caused by the environmental Gram-negative bacterium *Burkholderia pseudomallei* [1]. It is highly endemic in Southeast Asia and northern Australia [2], and has been increasingly reported across tropical regions worldwide, including Africa [3], Central and South America [4, 5] and, even in Mississippi, United States of America [6]. Naturally acquired infection results from exposure to *B. pseudomallei* via skin inoculation, inhalation or ingestion. The bacterium is intrinsically resistant to a wide range of antimicrobials commonly used for community-acquired sepsis and community-acquired pneumonia [1]. Effective parenteral antibiotics for acute melioidosis include ceftazidime and carbapenem drugs [7, 8]. Treatment with ineffective antimicrobials may result in case mortality exceeding 70% [9]. However, the mortality of culture-confirmed melioidosis patients in northern Australia has declined to lower than 10%, owing to early diagnosis, effective antimicrobial therapy and state-of-the-art intensive care [10]. The global burden of melioidosis is estimated at approximately 160,000 cases annually, resulting in around 89,000 deaths each year [2].

Several factors have been reported to be associated with the severity and mortality of melioidosis. These include host-related factors such as advanced age, underlying medical conditions (particularly diabetes mellitus, chronic kidney disease, and immunosuppression) [1], pathogen-related factors such as *bimA*_Bm_ gene (associated with neurological melioidosis) and *fhaB*3 gene (associated with bacteremia) [11], and healthcare-related factors such as timely diagnosis, appropriate antibiotic therapy, and access to and levels of intensive care support [12]. Among survivors, re-admission and recurrent melioidosis are also common [13].

Nonetheless, clinical factors associated with mortality remain inadequately evaluated because of variability in definitions of “severe melioidosis” [14] and the lack of multinational studies. The term “bacteremic melioidosis” is commonly used for distinguishing relatively more severe bacteremic patients from less severe non-bacteremic patients. However, bacteremia is identified only after bacterial culture results become available, and therefore cannot be used for patient stratification in routine clinical practice or prospective clinical trials. In addition, patients initially diagnosed with “suspected melioidosis” require empirical parenteral antibiotics recommended for melioidosis [10, 15, 16], yet this patient group is rarely evaluated systematically. A better understanding of factors associated with severity and mortality in both suspected and confirmed melioidosis would inform the design of future clinical trials aimed at reducing mortality among patients with melioidosis.

Here, we aim to prospectively evaluate the clinical epidemiology, mortality, factors associated with mortality, and other secondary outcomes among patients with suspected or confirmed melioidosis in Laos and Thailand. In addition, we systematically evaluate severity of sepsis at enrollment using the Sequential Organ Failure Assessment (SOFA) score [17].

## Materials and methods

### Ethics statement

We conducted the study in full compliance with the principles of good clinical practice (GCP), and the ethical principles of the Declaration of Helsinki. The study protocol and related documents were approved by the Ethics Committees of Srinagarind Hospital (HE661486), Sunpasitthiprasong Hospital (065/66C), the Faculty of Tropical Medicine, Mahidol University (MUTM 2023–075), the Lao National Ethics Committee for Health Research (88/NECHR), and the Oxford Tropical Research Ethics Committee at the University of Oxford (OXTREC 542–23). Written, informed consent was obtained from participants prior to enrollment.

### Study setting

From November 2023 to October 2024, we conducted a prospective observational study at

Mahosot Hospital (MH), Vientiane, Laos; Srinagarind Hospital (SRH), Khon Kaen, Thailand; and Sunpasitthiprasong Hospital (SUH), Ubon Ratchathani, Thailand. In 2023, Laos was classified as a lower-middle income country, with a health expenditure per capita of US$27, whereas Thailand was an upper-middle-income country, with a health expenditure per capita of US$327 [18]. MH is a university-affiliated tertiary-care hospital with 540 non-ICU beds and 60 ICU beds; SRH is a university-affiliated tertiary-care hospital with 1,025 non-ICU and 77 ICU beds, and SUH is a public tertiary-care hospital with 933 non-ICU beds and 255 ICU beds.

### Study design

We prospectively screened adult patients (age ≥ 18 years) with suspected or culture-confirmed melioidosis. Suspected-melioidosis cases were defined as hospitalized patients who fulfilled clinical criteria of community-acquired infection, were suspected of melioidosis, and were within 24 hours of hospitalization at the study hospitals. Confirmed-melioidosis cases were defined as patients who had a clinical specimen tested positive for *B. pseudomallei* by either culture or antigen-detection immunofluorescence microscopy (IFM).

Patients were defined as suspected melioidosis when they had septic shock, had sepsis with at least one risk factor associated with melioidosis (including diabetes mellitus, chronic kidney disease, thalassemia major, malignancy and immunosuppressive therapy) or were prescribed ceftazidime or meropenem by attending physicians. Because the study hospitals were in LMICs and all laboratory test results for defining the sequential organ failure assessment (SOFA) score may not have been available at the screening, patients in this study were classified as having sepsis if they had a SOFA score ≥2 or quick SOFA (qSOFA) score≥1 at screening. Septic shock was defined as sepsis requiring vasopressor therapy to maintain a mean arterial pressure ≥65 mmHg.

We excluded patients who were not of Thai or Lao nationality or were not admitted to the study hospitals. For suspected-melioidosis cases, we also excluded patients who had confirmed diagnoses of other infectious diseases, had non-infectious diseases suspected to be a primary cause of organ failure, were suspected of having hospital-acquired infections, or were transferred from other hospitals with a total duration of hospitalization >72 hours.

At the study hospitals, the study teams screened all medical patients by conducting ward rounds and reviewing admission logs in the emergency department, medical wards and medical ICUs daily. The study teams were also notified by healthcare workers in the emergency and other departments about potentially eligible patients. At screening, the study team assessed both qSOFA and SOFA scores, enrolling only participants who met the inclusion criteria and did not meet any exclusion criteria. Patients either presented directly to the study hospitals or were referred from other healthcare facilities. Confirmed-melioidosis cases were not necessarily identified at the time of admission; rather, the study teams were notified of positive *B. pseudomallei* culture results through the routine clinical microbiology laboratories of the study hospitals, and enrollment occurred during the patient’s hospitalization once the result became available.

After enrollment, the study teams conducted daily visits to monitor diagnostic test results, treatment received and clinical status until discharge from hospital or up to day 28 since enrollment, whichever occurred first. Day 1 was defined as the day of enrollment. On calendar days 28 and 90, the study team contacted participants by telephone to assess 28-day and 90-day mortality and other outcomes, including initiation of oral eradication treatment and hospital readmission.

The study did not involve any clinical interventions. All diagnostic tests and medical treatments were determined and prescribed by the attending physicians in accordance with standard-of-care. Of the three study hospitals, only SUH performed antigen-detection IFM as standard-of-care. The antigen-detection IFM was conducted as previously described [19]. In all three study hospitals, bacterial cultures were performed according to international standards, and organisms were identified using Matrix-Assisted Laser Desorption/Ionization Time-Of-Flight Mass Spectrometry. For patients enrolled in Laos, the cost of ceftazidime and meropenem was supported by the study.

### Outcome measures

The primary outcome measures were 28-day and 90-day mortality since enrollment. The secondary outcome measures included bacterial culture results, microbiology outcomes, time to blood culture negativity, duration of hospitalization (at the study hospital), antibiotic administration, use of mechanical ventilation, use of acute hemodialysis, hospital readmission and recurrent infection.

Follow-up blood culture sampling was defined as blood culture sampling on separate calendar days. Antibiotic administration was presented as Days of Therapy (DOTs), which was defined as the total number of calendar days on which a participant received at least one dose of an antibiotic, regardless of the dose and frequency of administration on that day. DOTs were counted separately for each antibiotic and then summed to give the total DOTs. For example, two different antibiotics prescribed on the same calendar day to a single patient, each contributed to one DOT, for a combined total of two DOTs. Length of Therapy (LOT) for ceftazidime or a carbapenem was defined as the total number of calendar days that a patient received at least one dose of ceftazidime, ceftazidime-avibactam, imipenem or meropenem regardless of the specific drug, the number of different drugs, the dose and frequency of administration. For example, ceftazidime and meropenem prescribed on the same calendar day to a single patient, both contributed to one LOT. The DOTs and LOT included the period from the first hospital admission (i.e., including the period at the transferring hospitals) to the first discharge at the study hospital. For antibiotics administered at transferring hospitals without a documented start date, the date of patient transfer was used as the start date of the antibiotic.

### Sample size

The sample size of the study was calculated using a simple precision-based approach to estimate the prevalence of a clinical characteristic or outcome among study population. A sample size of 200 was determined to provide 80% statistical power at a 5% alpha level, allowing for prevalence estimates with a 95% confidence interval width of ±10%.

### Statistical analysis

Medians, interquartile ranges (IQR) and ranges of continuous variables were estimated. IQRs are presented in terms of 25^th^ and 75^th^ percentiles. We compared proportions and continuous variables between groups using Fisher’s exact test and Kruskal-Wallis test, respectively. In the primary analysis, we used multivariable Cox proportional hazard models to evaluate factors associated with mortality risk within 90 days since enrollment. Patients who were lost to follow-up after hospital discharge were censored in the Cox proportional hazard models at the hospital discharge.

A total of five patients (of 43 patients) enrolled with suspected-melioidosis were subsequently diagnosed with culture-confirmed melioidosis after enrollment. In the main analysis, these five patients were included in the suspected-melioidosis group in order to describe the clinical characteristics and outcomes of patients enrolled in the suspected-melioidosis group. A sensitivity analysis was performed by reclassifying these five patients as culture-confirmed melioidosis patients. P values to two significant figures were provided, but not more than three decimal places. We used STATA (version 18.5; College Station, Texas) for the final statistical analysis.

## Results

### Baseline characteristics

**Fig 1:**
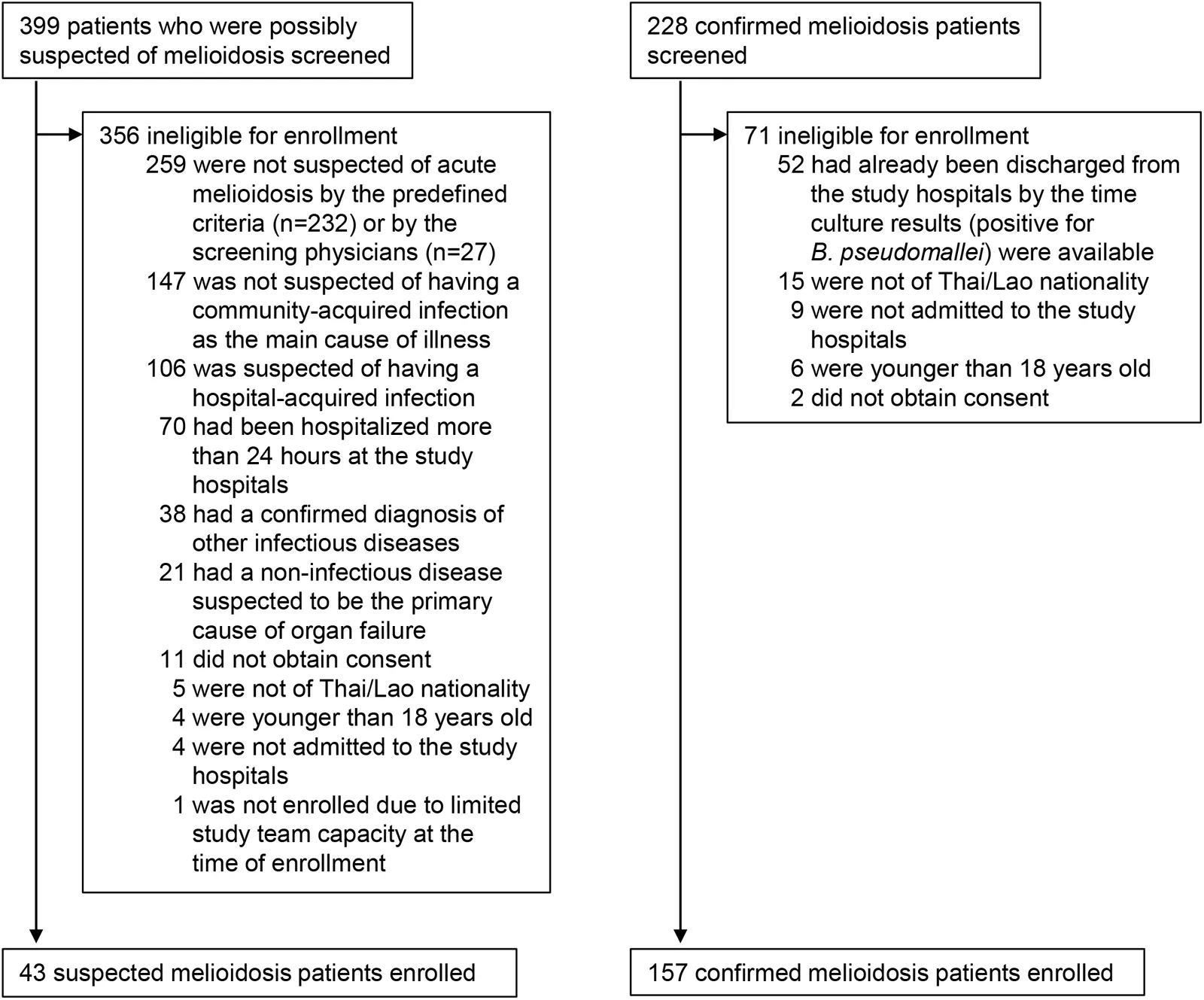
Flow diagram.

From November 2023 to October 2024, 399 patients were screened for suspected-melioidosis and 228 for confirmed-melioidosis (Fig 1). Of the 399 patients screened for suspected-melioidosis, 259 (65%) were excluded due to the absence of clinical suspicion that acute melioidosis was the main cause of illness. Of the 228 patients screened for confirmed-melioidosis, 52 (23%) were excluded because they had already been discharged from the study hospitals by the time culture results (positive for *B. pseudomallei*) were available. As these 52 patients were not enrolled in the study, their outcomes could not be assessed. Other common exclusion criteria included suspicion of having a hospital-acquired infection (n = 106), having a confirmed diagnosis of other infectious disease (n = 38), patients under 18 years of age (n = 10), patients who were not of Thai or Lao nationality (n = 20), those not admitted to the study hospitals (n = 13) and those from whom informed consent was not obtained (n = 13). In total, 43 suspected-melioidosis and 157 confirmed-melioidosis patients were enrolled in our study.

The baseline demographic and clinical characteristics of patients enrolled are detailed in Table 1. Of 200 patients, 136 (68%) were male, and the median age was 59 years (IQR 48–67 years, range 19–91 years). The most frequent underlying disease was diabetes mellitus (56%). The confirmed-melioidosis group had a higher proportion of transferred patients (53% vs. 21%, p < 0.001) and a longer median duration of admission at the study hospitals prior to enrollment (3 vs. 1 days, p < 0.001) compared to the suspected-melioidosis group.

**Table 1:** Baseline characteristics.

Characteristics	Suspected-melioidosis Group (n = 43)	Confirmed-melioidosis Group (n = 157)	P value
Study site			
Mahosot Hospital (MH)	5 (12%)	52 (33%)	<0.001
Srinagarind Hospital (SRH)	23 (53%)	9 (6%)	
Sunpasitthiprasong Hospital (SUH)	15 (35%)	96 (61%)	
Sex			
Male	27 (63%)	109 (69%)	0.46
Female	16 (37%)	48 (31%)	
Age, years	65 (56-76, 19-91)	57 (46-64, 19-91)	<0.001
Underlying diseases			
Diabetes mellitus	26 (60%)	86 (55%)	0.60
Hypertension	28 (65%)	50 (32%)	<0.001
Dyslipidemia	16 (37%)	24 (15%)	0.003
Heart disease	8 (19%)	11 (7%)	0.036
Liver disease	6 (14%)	6 (4%)	0.023
Chronic lung disease	10 (23%)	6 (4%)	<0.001
Chronic kidney disease	5 (12%)	12 (8%)	0.37
Major thalassemia	2 (5%)	2 (1%)	0.20
Malignancy	4 (9%)	8 (5%)	0.29
Immunosuppressive drug use	6 (14%)	7 (4%)	0.036
Previous melioidosis	3 (7%)	10 (6%)	>0.99
Transferred from other hospital	9 (21%)	83 (53%)	<0.001
Duration of admission at the study hospitals prior to enrollment (days)*	1 (0-1, 0-2)	3 (2-4, 0-30)	<0.001
Duration of symptoms prior to enrollment (days)			
<7	28 (65%)	40 (25%)	<0.001
7-13	7 (16%)	49 (31%)	
≥14	8 (19%)	68 (43%)	
Duration of effective parenteral antibiotics prior to enrollment (days)**	1 (0-1, 0-4)	2 (1-4, 0-23)	<0.001
SOFA score at enrollment			
0-1	17 (40%)	82 (52%)	0.22
2-3	6 (14%)	24 (15%)	
≥4	20 (47%)	51 (32%)	
In ICUs on the day of enrollment	11 (26%)	60 (38%)	0.15

Data are shown as n (%) or median (interquartile range, range). Previous melioidosis was defined as a documented history of a prior episode of either clinically diagnosed or culture-confirmed melioidosis. *A value of zero indicates enrollment on the admission date. Consent was obtained from three suspected-melioidosis patients on day three since hospital admission. **Effective antibiotics include ceftazidime, imipenem and meropenem. A value of zero indicates no effective parenteral antibiotics on the enrollment date (n = 9 patients). Duration includes effective parenteral antibiotics provided at transferring hospitals.

Of the 157 confirmed-melioidosis patients, 138 (88%) were enrolled following a positive culture result for *B. pseudomallei*, and 19 (12%) following a positive antigen-detection result using IFM. All 19 IFM-positive specimens were subsequently confirmed as culture-positive for *B. pseudomallei*. The median duration between the specimen collection date and the enrollment date was three days for bacterial culture and one day for IFM (p < 0.001). Six of the 19 patients (32%) enrolled following IFM results were enrolled on the same day their specimens were collected (i.e., duration = 0 day).

### Primary outcomes

**Fig 2:**
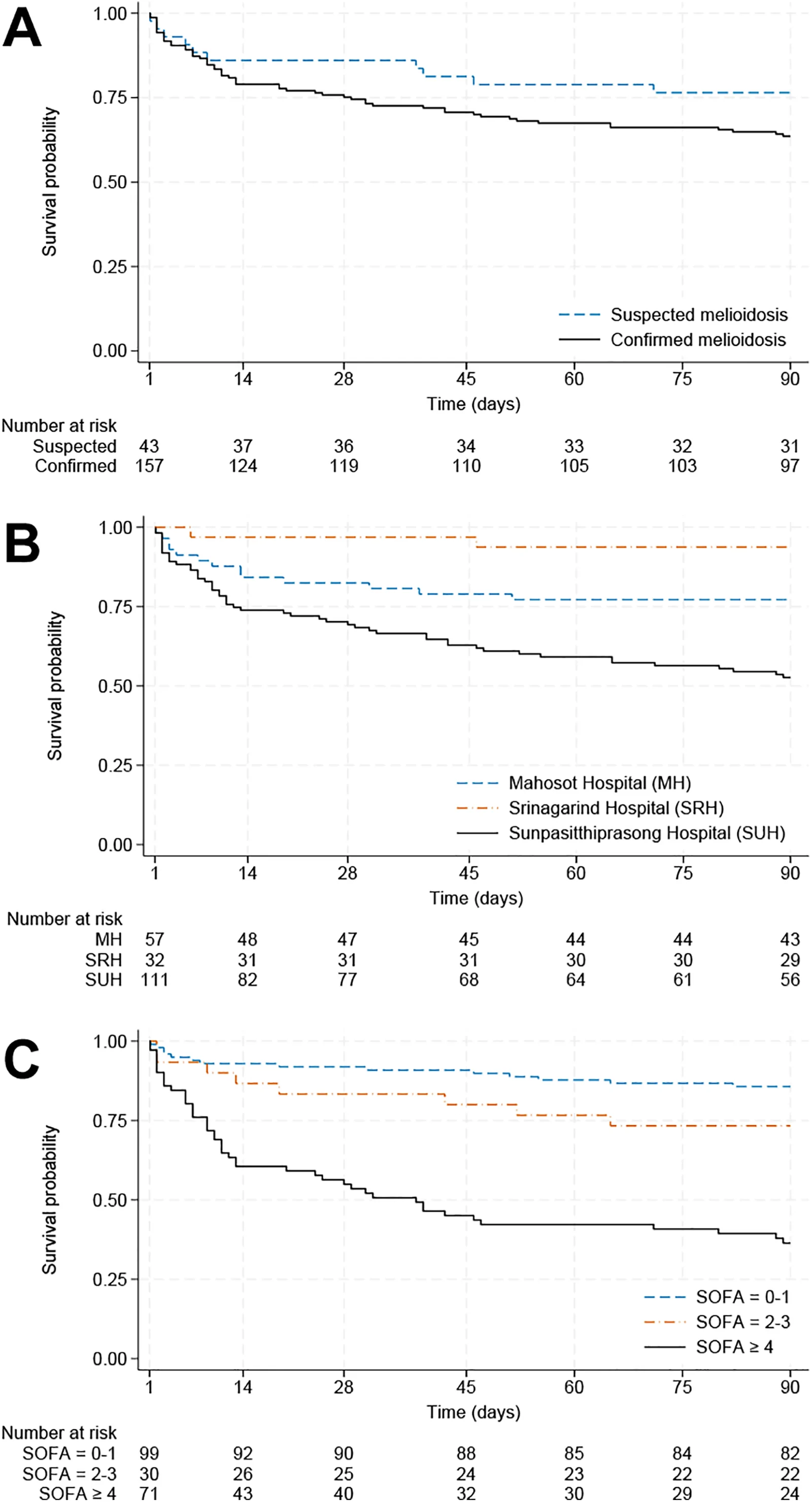
Kaplan-Meier plot analysis illustrating time to death since enrollment categorized by (A) suspected-melioidosis vs. confirmed-melioidosis patients, (B) study site, and (C) SOFA score at enrollment.

The overall 28-day and 90-day mortality was 14% (6/43) and 23% (10/43) in suspected-melioidosis patients, respectively; and 25% (39/157) and 36% (57/157) in confirmed-melioidosis patients, respectively (Table 2). In univariable Cox regression models, confirmed-melioidosis at enrollment, study site, male gender, higher SOFA score at enrollment, and ICU admission on the day of enrollment were associated with mortality outcomes (Fig 2 and Table 3). In multivariable Cox regression models, higher SOFA score at enrollment and ICU admission on the day of enrollment were strongly associated with mortality outcomes. Admission to SRH (adjusted hazard ratio 0.11; 95%CI 0.02-0.53, p = 0.003) and longer duration of symptoms prior to enrollment were independently associated with survival outcomes. Confirmed-melioidosis at enrollment was not independently associated with mortality outcomes.

**Table 2:** Outcomes.

Outcomes	Suspected-melioidosis group (n = 43)	Confirmed-melioidosis group (n = 157)
Mortality		
28-day mortality	6 (14%)	39 (25%)
90-day mortality	10 (23%)	57 (36%)
Lost to follow-up	1 (2%)	2 (1%)
Microbiology tests performed*		
Any clinical specimen	42 (98%)	157 (100%)
Blood culture	41 (95%)	150 (96%)
Respiratory tract specimen culture	28 (65%)	96 (61%)
Urine culture	28 (65%)	134 (85%)
Throat swab culture	3 (7%)	45 (29%)
Other clinical specimen culture	10 (23%)	71 (45%)
Microbiology outcomes**		
Any clinical specimen culture positive for *B. pseudomallei*	5 (12%)	157 (100%)
Blood specimen culture positive for *B. pseudomallei*	3 (7%)	93 (59%)
Respiratory tract specimen culture positive for *B. pseudomallei*	1 (2%)	57 (36%)
Urine specimen culture positive for *B. pseudomallei*	2 (5%)	30 (19%)
Throat swab culture positive for *B. pseudomallei*	–	19 (12%)
Other clinical specimen culture positive for *B. pseudomallei*	1 (2%)	45 (29%)
Median duration of hospitalization at the study hospitals	8 (5-16, 1-73)	15 (10-21, 2-92)
Mechanical ventilation*	14 (33%)	65 (41%)
Acute hemodialysis*	6 (14%)	24 (15%)
Transferred to other hospitals after discharge to continue receiving parenteral antibiotics	3 (7%)	37 (24%)
Readmissions because of worsening symptoms or new symptoms suspected to be associated with the cause of illness**	7 (16%)	17 (11%)

Data are shown as n (%) or median (interquartile range, range). *At any time prior to or since enrollment. No patients in the suspected-melioidosis group had a clinical specimen test result positive for *B. pseudomallei* by either culture-positive or antigen-detection using immunofluorescence microscopy (IFM) prior to enrollment. ** Within 90 days since enrollment**.** For each specimen type, the total number of clinical specimens culture positive for *B. pseudomallei* could be more than one per patient

**Table 3:** Factors associated with 90-day mortality.

Characteristics	Crude hazard ratio (95% CI)	P value	Adjusted hazard ratio (95% CI)	P value
Study group				
Suspected-melioidosis	1.0	0.12	1.0	0.59
Confirmed-melioidosis	1.65 (0.84-3.24)		1.22 (0.59-2.55)	
Study site				
Mahosot Hospital (MH)	1.0	<0.001	1.0	0.003
Srinagarind Hospital (SRH)	0.24 (0.06-1.08)		0.11 (0.02-0.53)	
Sunpasitthiprasong Hospital (SUH)	2.35 (1.28-4.32)		0.77 (0.36-1.62)	
Sex, men	1.94 (1.08-3.50)	0.028	–	–
Age, years	1.00 (0.98-1.01)	0.75	–	–
Diabetes mellitus	1.15 (0.71-1.88)	0.57	–	–
Transferred from other hospital	2.59 (1.56-4.29)	<0.001	–	–
Duration of symptoms prior to enrollment (days)				
<7	1.0	<0.001	1.0	0.034
7-13	1.23 (0.72-2.09)		0.91 (0.53-1.59)	–
≥14	0.36 (0.19-0.71)		0.43 (0.21-0.87)	–
Duration of effective parenteral antibiotics prior to enrollment (days)	0.99 (0.93-1.06)	0.77	–	–
SOFA score at enrollment				
0-1	1.0	<0.001	1.0	<0.001
2-3	1.99 (0.84-4.75)		1.99 (0.80-4.97)	
≥4	6.40 (3.50-11.7)		4.58 (2.01-10.44)	
In ICUs on the day of enrollment	4.33 (2.62-7.15)	<0.001	1.87 (1.02-3.41)	0.042

### Secondary outcomes

All patients had at least one clinical specimen collected for bacterial culture testing during the study period (Table 2). A total of 191 (96%), 124 (62%), 162 (81%), 48 (24%) and 81 (41%) patients had at least one blood, respiratory tract specimens, urine, throat swabs and other clinical specimen collected for bacterial culture, respectively. Of 43 patients enrolled in the suspected-melioidosis group, five were subsequently diagnosed with melioidosis through positive culture results from one or more of the following sample types: blood (n = 3 patients), respiratory tract specimen (n = 1 patient), urine (n = 2 patients) and other clinical specimen (n = 1 patient).

Follow-up blood cultures were frequently performed, but not systematically. Of the 96 patients with a blood culture positive for *B. pseudomallei*, 26 (27%) had no follow-up blood cultures, while 25 (26%), 10 (10%) and 35 (36%) had one, two, and at least three follow-up blood cultures, respectively. A total of 52 (54%) patients had at least one follow-up blood culture negative for *B. pseudomallei*, having a median time to blood culture negativity of 7 days (IQR 5–13 days, range 2–24 days) after the date of the first blood culture-positive specimen. Of the other 44 patients without follow-up blood culture-negative samples, 24 (55%) started oral eradication therapy without documented blood culture-negativity, 19 (43%) died during the study period before documented initiation of oral eradication therapy, and 1 (2%) was lost to follow-up after hospital discharge.

The median duration of hospitalization at the study hospitals was 15 days (IQR 9–21, range 1–92 days), and 43 patients (22%) were transferred to other hospitals after discharge to continue receiving parenteral antibiotics. During the study period, a total of 5,249 DOTs for parenteral antibiotics was prescribed (S1 Table). The most common parenteral antibiotic was ceftazidime (1,836 DOTs, 35%), followed by meropenem (1,138 DOTs, 22%). The median LOT for ceftazidime or a carbapenem was 6 days (IQR 3–14 days, range 0–46 days) in suspected-melioidosis group and 15 days (IQR 10–21 days, range 0–47 days) in confirmed-melioidosis group (p < 0.001, S1 Fig). A total of 79 patients (40%) received mechanical ventilation support and 30 patients (16%) received acute hemodialysis during the study period.

At day 90 since enrollment, of the 133 surviving patients, five (4%) remained hospitalized at the study hospitals, one (1%) was hospitalized at another hospital, and three (2%) could not be contacted after discharge from the study hospitals. Readmissions to either study hospitals or other hospitals because of worsening symptoms or new symptoms suspected to be associated with the cause of illness was noted in 24 (12%) of 200 patients. Based on daily visits by the study team prior to discharge and follow-up phone contact, prescription of oral eradication therapy was noted in 101 of 102 (99%) patients who had culture-confirmed melioidosis and could be contacted at day 90 since enrollment. Culture-confirmed recurrent melioidosis was not observed within the duration of the study.

### Sensitivity analysis

In the multivariable Cox regression models considering all patients with a clinical specimen culture positive for *B. pseudomallei* as culture-confirmed melioidosis, similar findings were observed with marginal difference in the 95%CIs.

## Discussion

In this multinational prospective observational study conducted in Laos and Thailand, we demonstrated that organ failure, as measured by the SOFA score, was strongly associated with mortality among suspected or confirmed melioidosis patients, and that the admitting study hospital was independently associated with survival outcomes. These findings are critical for designing future clinical trials aimed at reducing mortality of patients with suspected or confirmed melioidosis. Specifically, trials should consider stratification by both study site and severity of organ dysfunction at enrollment. In addition, sample size calculations based solely on data from one or few hospitals may lead to over or underestimation of a trial’s statistical power.

Our study highlights the high mortality of suspected-melioidosis patients. After adjusting for SOFA score, no difference in mortality was observed between patients with suspected or confirmed melioidosis. Our findings are consistent with previous randomized controlled trials (RCTs) where a difference in mortality between patients with and without melioidosis was not observed [9, 15, 20]. In one RCT [21], patients without melioidosis had an even higher mortality than those with melioidosis. This is possibly because patients who were enrolled in the suspected-melioidosis group in our study and in the previous RCTs had no known causes of sepsis at enrollment. However, this may differ when the causes of sepsis are known. In a large observational study [22], the mortality of melioidosis is relatively higher compared to other bacterial infections, such as *Staphylococcus aureus* and *Escherichia coli*, after adjusting for SOFA score.

The low mortality observed at SRH could be due to its relatively high level of intensive care support [23], consistent with findings from Australia where mortality of melioidosis patients has sharply declined following improvements in intensive care support [12, 24]. While access to ICUs, in our study, is associated with increased mortality, this likely represents an additional marker of disease severity in addition to the SOFA score. In all three study sites, diagnosis is based on bacterial culture, and treatment mainly involves ceftazidime and carbapenem drugs. These factors are adjusted for in the regression models. The relatively high mortality within 90 days observed at the other two study hospitals is consistent with the recent large observational study in public hospitals in Thailand [13].

The lower mortality of patients with a longer duration of symptoms is consistent with findings from previous studies where patients with chronic melioidosis (defined as symptoms present ≥2 months) [10] and those with a longer duration of symptoms [25] are associated with survival outcomes.

Our study has several strengths, including its multinational and prospective design, the systematic use of SOFA scores at enrollment, financial support for ceftazidime and meropenem at the study hospital in a lower-middle income country, daily monitoring of diagnosis, treatment and clinical status up to the 28-day hospitalization period, and high rates of follow-up for 28-day and 90-day outcomes. There are also several limitations. First, this is an observational study and multiple specific tests were not always available or conducted (e.g., arterial blood gas) within 24–48 hours of enrollment due to a lack of resources. This could lead to an underestimate of the SOFA scores. Time to blood culture negativity was not determined using time-to-event models because follow-up blood cultures were not performed systematically. Attending physicians could start oral eradication therapy once patients had completed parenteral antibiotic regimens and had fever clearance, without follow-up blood culture. Second, our prospective study was unable to systematically assess outcomes of culture-confirmed patients who were not enrolled (n = 52). Most of these patients died before screening (personal observation). Therefore, within the culture-confirmed melioidosis group, our findings are subject to survivor bias, as the study patients were limited to those who survived until culture results became available. Third, the relatively small sample size may limit statistical power to detect associations with modest effect sizes. Fourth, our follow-up was limited to 90 days, and lacked sufficient duration to assess recurrent melioidosis, as relapse commonly occurs within 1–2 years after the initiation of oral eradication treatment [26, 27]. Fifth, 28-day and 90-day follow-up was via telephone interviews, and the information obtained from patients or their relatives was subject to recall bias or incomplete reporting.

## Conclusion

In our prospective multinational observational study of adults with suspected or confirmed melioidosis, we found that their mortality remains high and varies according to the severity of organ dysfunction at enrollment, the duration of symptoms prior to enrollment, ICU admission on the day of enrollment, and the study hospitals. The lower mortality observed at SRH, after adjusting for confounding factors, suggests that enhancing levels of intensive care support could be one of the key factors in reducing mortality from sepsis and melioidosis in LMICs. Furthermore, the design of new RCTs should ensure that efficacy and effectiveness of candidate antibiotics can be robustly assessed under these conditions and as per international regulations.

### Conflict of interest

The authors declare that the research was conducted in the absence of any commercial or financial relationships that could be construed as a potential conflict of interest.

## Supporting information

S1 Table Days of Therapy (DOTs) for each parenteral antibiotic prescribed in 200 patients included in the study. (DOCX)

S1 Fig Median Length of Therapy (LOT) for ceftazidime or a carbapenem in 200 patients included in the study. (DOCX)
