# Supplementary material for: Outcomes of patients with suspected or confirmed melioidosis: A prospective multinational, multicenter, observational study
Source: PLoS Negl Trop Dis. 2026 Sep 8;20(9):e0014562. doi: 10.1371/journal.pntd.0014562 (PMC13577467; doi:10.1371/journal.pntd.0014562)
Supplement: Supplementary file 1 [file pntd.0014562.s001.docx]

**S1** **Table.** **Days of Therapy (DOTs) for each parenteral antibiotic prescribed in 200 patients included in the study**

| **Antibiotics** | Suspected-melioidosis group  (Total DOTs=709) | Confirmed-melioidosis group  (Total DOTs=4,540) |
| --- | --- | --- |
| Ceftazidime | 270 (38%) | 1566 (34%) |
| Meropenem | 117 (17%) | 1021 (22%) |
| Colistin | 36 (5%) | 306 (7%) |
| Ceftriaxone | 68 (10%) | 259 (6%) |
| Clindamycin | 27 (4%) | 256 (6%) |
| Metronidazole | 24 (3%) | 158 (3%) |
| Vancomycin | 15 (2%) | 133 (3%) |
| Sulbactam | 12 (2%) | 135 (3%) |
| Cefoperazone/sulbactam | 0 (0%) | 106 (2%) |
| Piperacillin/tazobactam | 30 (4%) | 64 (1%) |
| Fosfomycin | 5 (1%) | 88 (2%) |
| Azithromycin | 23 (3%) | 63 (1%) |
| Trimethoprim/sulfamethoxazole | 5 (1%) | 67 (1%) |
| Cefazolin | 5 (1%) | 46 (1%) |
| Tigecycline | 20 (3%) | 28 (1%) |
| Levofloxacin | 9 (1%) | 36 (1%) |
| Ciprofloxacin | 15 (2%) | 23 (1%) |
| Gentamicin | 0 (0%) | 37 (1%) |
| Penicillin G | 3 (0%) | 37 (1%) |
| Cloxacillin | 4 (1%) | 20 (0%) |
| Imipenem | 0 (0%) | 24 (1%) |
| Amoxicillin/clavulanic acid | 5 (1%) | 12 (0%) |
| Ceftazidime/avibactam | 0 (0%) | 17 (0%) |
| Amikacin | 1 (0%) | 11 (0%) |
| Cefotaxime | 11 (2%) | 0 (0%) |
| Ampicillin/sulbactam | 0 (0%) | 9 (0%) |
| Ampicillin | 0 (0%) | 8 (0%) |
| Ertapenem | 4 (1%) | 3 (0%) |
| Streptomycin | 0 (0%) | 6 (0%) |
| Cefoperazone | 0 (0%) | 1 (0%) |

Footnote: DOTs were defined as the total number of calendar days that a patient was prescribed an antibiotic regardless of the dose and frequency of administration. DOTs were counted separately for each antibiotic and then summed to give the total DOTs. For example, two different antibiotics prescribed on the same calendar day to a single patient, each contributed to one DOT, for a combined total of two DOTs. The DOTs included the period from the first hospital admission (i.e. including DOTs at the transferring hospitals) to the first discharge at the study hospital. The total number of patients was 43 in the suspected-melioidosis group, and 157 in the confirmed melioidosis group.
