# Supplementary material for: Outcomes of patients with suspected or confirmed melioidosis: A prospective multinational, multicenter, observational study
Source: PLoS Negl Trop Dis. 2026 Sep 8;20(9):e0014562. doi: 10.1371/journal.pntd.0014562 (PMC13577467; doi:10.1371/journal.pntd.0014562)
Supplement: Supplementary file 2 [file pntd.0014562.s002.docx]

**S1 Fig.** **Median Length of Therapy (LOT) for ceftazidime or a carbapenem in 200 patients included in the study**

Footnote: E=Enrollment day. Data are shown as median (interquartile range, range). LOT for ceftazidime (including ceftazidime and ceftazidime-avibactam) or a carbapenem (including meropenem and imipenem) was defined as the total number of calendar days that a patient was prescribed either ceftazidime or a carbapenem drug regardless of the specific drug, the dose and frequency of administration. For example, ceftazidime and meropenem prescribed on the same calendar day to a single patient, counted as one LOT. The LOT is presented as the median (interquartile range, and range). The LOT included the period from the first hospital admission (i.e. including LOT at the transferring hospitals) to the first discharge at the study hospital. The total number of patients was 43 in the suspected-melioidosis group, and 157 in the confirmed melioidosis group.
