# Supplementary material for: Outcomes of patients with suspected or confirmed melioidosis: A prospective multinational, multicenter, observational study
Source: PLoS Negl Trop Dis. 2026 Sep 8;20(9):e0014562. doi: 10.1371/journal.pntd.0014562 (PMC13577467; doi:10.1371/journal.pntd.0014562)
Supplement: Supplementary file 3 [file pntd.0014562.s003.docx]

*Re: Decision on your PLOS Neglected Tropical Diseases submission (PNTD-D-26-00365) - [EMID:59fd48a58f8747e4]*

*XX July 2026*

*Dear Dr. Picardeau, Dr. Kamhawi and Dr. Brindley, and Reviewers,*

*Thank you for your e-mail regarding the above manuscript. We are grateful for the helpful comments made by the editors and reviewers, and have addressed them as detailed below.*

**Methods**

**Reviewer #1: Methods were clearly stated and appropriate for the overall study, although sample size of subset of suspected melioidosis patients was smaller than one would have hoped, especially when comparing suspect vs confirmed cases. Better design would have included equal numbers of suspected and confirmed, but for the limited analysis carried out in this study, this probably was not an issue. Statistical analysis was appropriate, and study population clearly defined.**

*Response: We are grateful for the comment.*

**Reviewer #2: The sample size was small, and the authors acknowledged this in the discussion section. Was the sample size limited by the duration of the study?**

*Response: The target sample size of 200 was pre-specified and was reached within the planned 12-month enrollment period; the duration of the study was therefore not the factor that limited the total sample size.*

**Results**

**Reviewer #1: Results were clearly stated, although as mentioned above, sample size for subset of suspected melioidosis cases was smaller than ideal. Figures are appropriate and clear, covering all important results and findings.**

**Reviewer #2: (No Response)**

*Response: We are grateful for the comment.*

**Conclusions**

**Reviewer #1: Yes, conclusions were supported and strengths and weaknesses clearly stated. They do discuss the importance of further studies to further elucidate factors related to mortality, as well as the limitations presented by following patients for only 90 days. Public health relevance is addressed as well.**

**Reviewer #2: (No Response)**

*Response: We are grateful for the comment.*

**Editorial and Data Presentation Modifications?**

**Reviewer #1: I do not note any changes or editorial suggestions; manuscript was clear in my opinion and does not require modification.**

**Reviewer #2: (No Response)**

*Response: We are grateful for the comment.*

**Summary and General Comments**

**Reviewer #1: (No Response)**

**Reviewer #2: This multicenter study in tertiary hospitals in Laos (1 university-affiliated) and Thailand (1 university-affiliated and 1 public) examined outcomes among hospitalized adults with suspected or confirmed melioidosis. Patients with confirmed melioidosis had high death rates within 28 and 90 days. Severe illness at hospital admission, especially organ failure and ICU admission, was strongly linked to poorer outcomes. Given that this is an observational study, the data speak for themselves. However, the manuscript and its important messages can be made much clearer with improved English.**

*Response: The manuscript has been revised to improve clarity of the English.*

**Points/Queries:**

**1. Regarding enrollment of study participants, were all the patients referred from primary and secondary health facilities? This query arose because, based on the manuscript description, it was not clear how the melioidosis status of some patients could be immediately confirmed upon admission at the three study sites (hospitals).**

*Response: All patients were enrolled at the study hospitals. Some patients were referred from primary or secondary health facilities to the study hospitals. The status of both suspected and confirmed-melioidosis patients was confirmed at the study hospitals. The following sentences have been added for clarity, “Patients either presented directly to the study hospitals or were referred from other healthcare facilities. Confirmed-melioidosis cases were not necessarily identified at the time of admission; rather, the study teams were notified of positive B. pseudomallei culture results through the routine clinical microbiology laboratories of the study hospitals, and enrollment occurred during the patient’s hospitalization once the result became available.”*

**2. Line 106 & 108: Please specify the currency of the health expenditure per capita. Please also cite the source of the information.**

*Response: Revised as suggested.*

**3. Table 1:**

**i) Only the number of male participants was presented. Were female patients excluded from the study?**

*Response: The numbers of female participants have been added for clarity.*

**ii) What does “Previous melioidosis” mean in this study?**

*Response: “Previous melioidosis was defined as a documented history of a prior episode of either clinically diagnosed or culture-confirmed melioidosis.” The sentence has been added for clarity.*

**4. Based on the definition given by the authors, DOTs and LOT are actually the same, referring to the “total number of calendar days” patients were prescribed an antibiotic. It is suggested that a single term or phrase be used for greater clarity.**

*Response: Due to the differences between DOTs and LOT, and the distinct advantages each offers, we have retained both metrics. For clarity, we have added explanatory sentences to both the Methods section and the Appendix.*

**Journal Requirements:**

**1) Please ensure that the CRediT author contributions listed for every co-author are completed accurately and in full.**

**At this stage, the following Authors/Authors require contributions: Direk Limmathurotsakul, Koukeo Phommasone, Siriluck Anunnatsiri, Suwatthiya Kitsaran, Sue J Lee, Pankham Vongphachanh, Ko Chang, George H Talbot, Kevin O Shea, Paul B Eckburg, Stephen D Prior, Elizabeth A Ashley, and Nicholas PJ Day. Please ensure that the full contributions of each author are acknowledged in the "Add/Edit/Remove Authors" section of our submission form.**

**The list of CRediT author contributions may be found here:** [**https://journals.plos.org/plosntds/s/authorship#loc-author-contributions**](https://journals.plos.org/plosntds/s/authorship#loc-author-contributions)

*Response: Revised as suggested.*

**2) Please provide an Author Summary. This should appear in your manuscript between the Abstract (if applicable) and the Introduction, and should be 150-200 words long. The aim should be to make your findings accessible to a wide audience that includes both scientists and non-scientists. Sample summaries can be found on our website under Submission Guidelines:**

[**https://journals.plos.org/plosntds/s/submission-guidelines#loc-parts-of-a-submission**](https://journals.plos.org/plosntds/s/submission-guidelines#loc-parts-of-a-submission)

*Response: Revised as suggested.*

**3) In the online submission form, you indicated that All relevant data are within the manuscript and its Supporting Information files. De-identified individual participant data are available upon reasonable request to the corresponding author, subject to approval by the institutional ethics committee.. All PLOS journals now require all data underlying the findings described in their manuscript to be freely available to other researchers, either**

**1. In a public repository**

**2. Within the manuscript itself**

**3. Uploaded as supplementary information.**

**This policy applies to all data except where public deposition would breach compliance with the protocol approved by your research ethics board. If your data cannot be made publicly available for ethical or legal reasons (e.g., public availability would compromise patient privacy), please explain your reasons by return email and your exemption request will be escalated to the editor for approval. Your exemption request will be handled independently and will not hold up the peer review process, but will need to be resolved should your manuscript be accepted for publication. One of the Editorial team will then be in touch if there are any issues.**

*Response: We confirmed that “All relevant data are within the manuscript and its Supporting Information files. De-identified individual participant data are available upon reasonable request to the corresponding author, subject to approval by the institutional ethics committee.” Uploading the de-identified individual participant data in a public repository would breach compliance with the protocol approved by our research ethics board.*

*All contributing authors have reviewed and concurred with the revised manuscript. The funders had no role in study design, data collection and analysis, decision to publish, or preparation of the manuscript.*

*We would like to thank you again for the helpful comments and hope the manuscript is now acceptable for publication in the PLOS Neglected Tropical Diseases*

*Sincerely,*

*Direk Limmathurotsakul, MD, MSc, PhD*

*Corresponding author*

*Head of Microbiology*

*Mahidol-Oxford Tropical Medicine Research Unit*

*E-mail: direk@tropmedres.ac*
