# Supplementary material for: Outcomes of patients with suspected or confirmed melioidosis: A prospective multinational, multicenter, observational study
Source: PLoS Negl Trop Dis. 2026 Sep 8;20(9):e0014562. doi: 10.1371/journal.pntd.0014562 (PMC13577467; doi:10.1371/journal.pntd.0014562)
Supplement: Peer review history

# Peer review: Decision Letter 0

**DOI:** 10.1371/journal.pntd.0014562.r001

**Authors:** Mathieu Picardeau

18 Jun 2026

Response to Reviewers Revised Manuscript with Track Changes Manuscript

Shaden Kamhawi

co-Editor-in-Chief

PLOS Neglected Tropical Diseases

orcid.org/0000-0003-4304-636XX

Paul Brindley

co-Editor-in-Chief

PLOS Neglected Tropical Diseases

orcid.org/0000-0003-1765-0002

**Additional Editor Comments (if provided):** **Journal Requirements:**

1) Please ensure that the CRediT author contributions listed for every co-author are completed accurately and in full.

At this stage, the following Authors/Authors require contributions: Direk Limmathurotsakul, Koukeo Phommasone, Siriluck Anunnatsiri, Suwatthiya Kitsaran, Sue J Lee, Pankham Vongphachanh, Ko Chang, George H Talbot, Kevin O Shea, Paul B Eckburg, Stephen D Prior, Elizabeth A Ashley, and Nicholas PJ Day. Please ensure that the full contributions of each author are acknowledged in the "Add/Edit/Remove Authors" section of our submission form.

The list of CRediT author contributions may be found here: https://journals.plos.org/plosntds/s/authorship#loc-author-contributions

2) Please provide an Author Summary. This should appear in your manuscript between the Abstract (if applicable) and the Introduction, and should be 150-200 words long. The aim should be to make your findings accessible to a wide audience that includes both scientists and non-scientists. Sample summaries can be found on our website under Submission Guidelines:

https://journals.plos.org/plosntds/s/submission-guidelines#loc-parts-of-a-submission

3) In the online submission form, you indicated that All relevant data are within the manuscript and its Supporting Information files. De-identified individual participant data are available upon reasonable request to the corresponding author, subject to approval by the institutional ethics committee.. All PLOS journals now require all data underlying the findings described in their manuscript to be freely available to other researchers, either

1. In a public repository

2. Within the manuscript itself

3. Uploaded as supplementary information.

This policy applies to all data except where public deposition would breach compliance with the protocol approved by your research ethics board. If your data cannot be made publicly available for ethical or legal reasons (e.g., public availability would compromise patient privacy), please explain your reasons by return email and your exemption request will be escalated to the editor for approval. Your exemption request will be handled independently and will not hold up the peer review process, but will need to be resolved should your manuscript be accepted for publication. One of the Editorial team will then be in touch if there are any issues.

**Reviewers' comments:**

**Key Review Criteria Required for Acceptance?**

As you describe the new analyses required for acceptance, please consider the following:

**Methods**

-Are the objectives of the study clearly articulated with a clear testable hypothesis stated?

-Is the study design appropriate to address the stated objectives?

-Is the population clearly described and appropriate for the hypothesis being tested?

-Is the sample size sufficient to ensure adequate power to address the hypothesis being tested?

-Were correct statistical analysis used to support conclusions?

-Are there concerns about ethical or regulatory requirements being met?

Reviewer #1: Methods were clearly stated and appropriate for the overall study, although sample size of subset of suspected melioidosis patients was smaller than one would have hoped, especially when comparing suspect vs confirmed cases. Better design would have included equal numbers of suspected and confirmed, but for the limited analysis carried out in this study, this probably was not an issue. Statistical analysis was appropriate, and study population clearly defined.

Reviewer #2: The sample size was small, and the authors acknowledged this in the discussion section. Was the sample size limited by the duration of the study?

**********

**Results**

-Does the analysis presented match the analysis plan?

-Are the results clearly and completely presented?

-Are the figures (Tables, Images) of sufficient quality for clarity?

Reviewer #1: Results were clearly stated, although as mentioned above, sample size for subset of suspected melioidosis cases was smaller than ideal. Figures are appropriate and clear, covering all important results and findings.

Reviewer #2: (No Response)

**********

**Conclusions**

-Are the conclusions supported by the data presented?

-Are the limitations of analysis clearly described?

-Do the authors discuss how these data can be helpful to advance our understanding of the topic under study?

-Is public health relevance addressed?

Reviewer #1: Yes, conclusions were supported and strengths and weaknesses clearly stated. They do discuss the importance of further studies to further elucidate factors related to mortality, as well as the limitations presented by following patients for only 90 days. Public health relevance is addressed as well.

Reviewer #2: (No Response)

**********

**Editorial and Data Presentation Modifications?**

Use this section for editorial suggestions as well as relatively minor modifications of existing data that would enhance clarity. If the only modifications needed are minor and/or editorial, you may wish to recommend “Minor Revision” or “Accept”.

Reviewer #1: I do not note any changes or editorial suggestions; manuscript was clear in my opinion and does not require modification.

Reviewer #2: (No Response)

**********

**Summary and General Comments**

Use this section to provide overall comments, discuss strengths/weaknesses of the study, novelty, significance, general execution and scholarship. You may also include additional comments for the author, including concerns about dual publication, research ethics, or publication ethics. If requesting major revision, please articulate the new experiments that are needed.

Reviewer #1: (No Response)

Reviewer #2: This multicenter study in tertiary hospitals in Laos (1 university-affiliated) and Thailand (1 university-affiliated and 1 public) examined outcomes among hospitalized adults with suspected or confirmed melioidosis. Patients with confirmed melioidosis had high death rates within 28 and 90 days. Severe illness at hospital admission, especially organ failure and ICU admission, was strongly linked to poorer outcomes. Given that this is an observational study, the data speak for themselves. However, the manuscript and its important messages can be made much clearer with improved English.

Points/Queries:

1. Regarding enrollment of study participants, were all the patients referred from primary and secondary health facilities? This query arose because, based on the manuscript description, it was not clear how the melioidosis status of some patients could be immediately confirmed upon admission at the three study sites (hospitals).

2. Line 106 & 108: Please specify the currency of the health expenditure per capita. Please also cite the source of the information.

3. Table 1:

i) Only the number of male participants was presented. Were female patients excluded from the study?

ii) What does “Previous melioidosis” mean in this study?

4. Based on the definition given by the authors, DOTs and LOT are actually the same, referring to the “total number of calendar days” patients were prescribed an antibiotic. It is suggested that a single term or phrase be used for greater clarity.

**********

PLOS authors have the option to publish the peer review history of their article (what does this mean?). If published, this will include your full peer review and any attached files.

If you choose “no”, your identity will remain anonymous but your review may still be made public.

**Do you want your identity to be public for this peer review?**  For information about this choice, including consent withdrawal, please see our Privacy Policy.

Reviewer #1: **Yes:** David D Blaney, MD MPH FACPM

Reviewer #2: No

**Figure resubmission:**

**Reproducibility:**

To enhance the reproducibility of your results, we recommend that authors of applicable studies deposit laboratory protocols in protocols.io, where a protocol can be assigned its own identifier (DOI) such that it can be cited independently in the future. Additionally, PLOS ONE offers an option to publish peer-reviewed clinical study protocols. Read more information on sharing protocols at https://plos.org/protocols?utm_medium=editorial-email&utm_source=authorletters&utm_campaign=protocols

# Author comment: Author response to Decision Letter 1

**DOI:** 10.1371/journal.pntd.0014562.r002

7 Jul 2026

Attachment Submitted filename: Response to Reviewers.docx

# Editor report: Decision Letter 1

**DOI:** 10.1371/journal.pntd.0014562.r003

**Authors:** Mathieu Picardeau

9 Jul 2026

Dear Limmathurotsakul,

We are pleased to inform you that your manuscript 'Outcomes of patients with suspected or confirmed melioidosis: a prospective multi-national, multicenter, observational study' has been provisionally accepted for publication in PLOS Neglected Tropical Diseases.

Before your manuscript can be formally accepted you will need to complete some formatting changes, which you will receive in a follow up email. A member of our team will be in touch with a set of requests.

Please note that your manuscript will not be scheduled for publication until you have made the required changes, so a swift response is appreciated.

IMPORTANT: The editorial review process is now complete. PLOS will only permit corrections to spelling, formatting or significant scientific errors from this point onwards. Requests for major changes, or any which affect the scientific understanding of your work, will cause delays to the publication date of your manuscript.

Should you, your institution's press office or the journal office choose to press release your paper, you will automatically be opted out of early publication. We ask that you notify us now if you or your institution is planning to press release the article. All press must be co-ordinated with PLOS.

Thank you again for supporting Open Access publishing; we are looking forward to publishing your work in PLOS Neglected Tropical Diseases.

Best regards,

Mathieu Picardeau

Section Editor

PLOS Neglected Tropical Diseases

Shaden Kamhawi

co-Editor-in-Chief

PLOS Neglected Tropical Diseases

orcid.org/0000-0003-4304-636XX

Paul Brindley

co-Editor-in-Chief

PLOS Neglected Tropical Diseases

orcid.org/0000-0003-1765-0002

***********************************************************

p.p1 {margin: 0.0px 0.0px 0.0px 0.0px; line-height: 16.0px; font: 14.0px Arial; color: #323333; -webkit-text-stroke: #323333}span.s1 {font-kerning: none

# Editor report: Acceptance letter

**DOI:** 10.1371/journal.pntd.0014562.r004

**Authors:** Mathieu Picardeau

Dear Limmathurotsakul,

We are delighted to inform you that your manuscript, "Outcomes of patients with suspected or confirmed melioidosis: a prospective multi-national, multicenter, observational study," has been formally accepted for publication in PLOS Neglected Tropical Diseases.

We have now passed your article onto the PLOS Production Department who will complete the rest of the publication process. All authors will receive a confirmation email upon publication.

The corresponding author will soon be receiving a typeset proof for review, to ensure errors have not been introduced during production. Please review the PDF proof of your manuscript carefully, as this is the last chance to correct any scientific or type-setting errors. Please note that major changes, or those which affect the scientific understanding of the work, will likely cause delays to the publication date of your manuscript. Note: Proofs for Front Matter articles (Editorial, Viewpoint, Symposium, Review, etc...) are generated on a different schedule and may not be made available as quickly.

Soon after your final files are uploaded, the early version of your manuscript will be published online unless you opted out of this process. The date of the early version will be your article's publication date. The final article will be published to the same URL, and all versions of the paper will be accessible to readers.

For Research Articles, you will receive an invoice from PLOS for your publication fee after your manuscript has reached the completed accept phase. If you receive an email requesting payment before acceptance or for any other service, this may be a phishing scheme. Learn how to identify phishing emails and protect your accounts at https://explore.plos.org/phishing.

Thank you again for supporting open-access publishing; we are looking forward to publishing your work in PLOS Neglected Tropical Diseases.

Best regards,

Shaden Kamhawi

co-Editor-in-Chief

PLOS Neglected Tropical Diseases

Paul Brindley

co-Editor-in-Chief

PLOS Neglected Tropical Diseases
